# On the decline of biodiversity due to area loss

**DOI:** 10.1038/ncomms9837

**Published:** 2015-11-17

**Authors:** Petr Keil, David Storch, Walter Jetz

**Affiliations:** 1 Department of Ecology and Evolutionary Biology, Yale University, 165 Prospect Street, New Haven, Connecticut 06520-8106, USA; 2 Center for Theoretical Study, Jilská 1, 110 00, Prague 1, Czech Republic; 3 Department of Ecology, Faculty of Science, Charles University, Viničná 7, 128 44, Prague 2, Czech Republic

## Abstract

Predictions of how different facets of biodiversity decline with habitat loss are broadly needed, yet challenging. Here we provide theory and a global empirical evaluation to address this challenge. We show that extinction estimates based on endemics–area and backward species–area relationships are complementary, and the crucial difference comprises the geometry of area loss. Across three taxa on four continents, the relative loss of species, and of phylogenetic and functional diversity, is highest when habitable area disappears inward from the edge of a region, lower when it disappears from the centre outwards, and lowest when area is lost at random. In inward destruction, species loss is almost proportional to area loss, although the decline in phylogenetic and functional diversity is less severe. These trends are explained by the geometry of species ranges and the shape of phylogenetic and functional trees, which may allow baseline predictions of biodiversity decline for underexplored taxa.

## Abstract (web summary)

Habitat loss typically results in biodiversity decline, yet predicting how different facets of biodiversity are affected is challenging. Here, the authors show that the geometry of area loss is crucial to predict loss of taxonomic, functional and phylogenetic diversity across three taxa on four continents.

## Graphical abstract

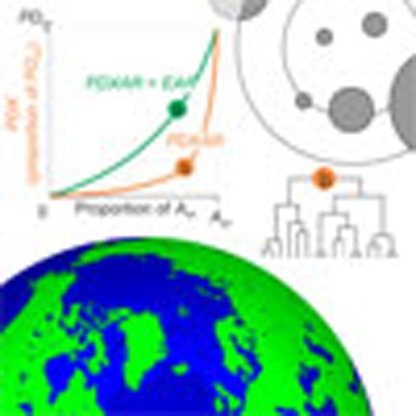

Habitat loss due to accelerated climate change and direct human impact has been causing a decline of biodiversity and associated ecosystem services^1^,^2^,^3^. Direct estimates of biodiversity loss are challenging because of highly incomplete global species' distribution knowledge^4^ and the difficulties of ascertaining actual extinctions^5^,^6^,^7^. Instead, estimates of diversity loss have relied on indirect methods, such as the relationship between area and the number of species in that area, the species–area relationship (SAR)^8^,^9^,^10^,^11^, or the relationship between an area that is lost and the number of species confined to it, the endemics–area relationship (EAR)^12^,^13^.

Recently, He and Hubbell^12^ initiated discussion over the reliability of the indirect methods, claiming that the SAR-based method (also known as backward estimation) overestimates extinctions when compared with the EAR-based method (forward estimation). This debate has generated several valuable insights, such as recognition that EAR and SAR are linked by the complementarity of area that is lost and the area that remains^14^, recognition of point reflection symmetry of the two curves^13^, or identification of critical role of aggregation of individuals^15^ and ecological context^16^,^17^ at small-scale plots. However, this debate has not yet been settled, and there are still critical unresolved issues, as well as opportunities for synthesis.

Specifically, it has been suggested that different spatial arrangements of habitat loss lead to different extinction estimates^18^,^19^,^20^,^21^, which follows implicitly from comparing estimates of the forward and backward methods^8^,^12^,^16^. The emerging pattern has been that, given the same amount of lost habitable area, inward area loss starting on the edges of a region leads to higher average proportional loss of species richness than when area is lost outward from within the centre of the region. Yet, it is unclear if such pattern is inevitable, that is, if there is a theoretical possibility that, contrary to He and Hubbell's claim^12^, SARs (the backward method) can also actually underestimate extinction rates. Further, apart from the anecdotal case of US birds^12^, this discrepancy has been demonstrated, and explained by aggregation of individuals^15^, only at small scales. It is unknown if the discrepancy holds at continental to global scales, and if it holds, what generates it. These scales are critical, as the whole species' ranges are lost at such large scales, and the extinctions are thus irreversible. Also, it is the global scale at which the current high-profile debate on the magnitude of diversity loss (that is, extinction crisis) takes place^5^,^22^. In contrast, studies of diversity loss with area loss have been mostly confined to local plots^12^, which have conservation relevance in local context, but are irrelevant for global estimates (see Supplementary Note 1 for details). In addition, both the existing theory and empirical assessments of diversity loss under area loss have traditionally comprised only the number of species, discounting often highly variable evolutionary and functional uniqueness of species^23^.

Here we overcome these limitations by explicitly addressing how the spatial configuration of area loss affects the loss of species, and of associated phylogenetic diversity (PD) and functional diversity (FD)^24^,^25^. In the first part, we advance the theoretical basis for the estimation of the decline of taxonomic, phylogenetic and functional diversity due to area loss, with particular emphasis on geometry of the area loss. We then empirically address these issues using data on three major vertebrate taxa in nine large-scale regions and three geometries of area loss: contiguous inward, outward and randomly scattered. We further relate the magnitude of diversity loss to predictors such as mean range size, range clumpedness and shape. We show that the commonly used extinction estimates based purely on area loss are misleading at large scales—the direction of the area loss is crucial, with a contiguous area loss coming from the edges of regions inwards being, on average, the most serious threat to biodiversity. Second, the direction of area loss is consistently more important for extinction estimates than mean species range size. Third, PD and FD are more resistant to loss of area than species richness. Finally, we show how this resistance is related to taxon-wide estimates of phylogenetic and functional similarity.

## Results

### Geometry of area loss and its links to SAR and EAR

**Figure 1:**
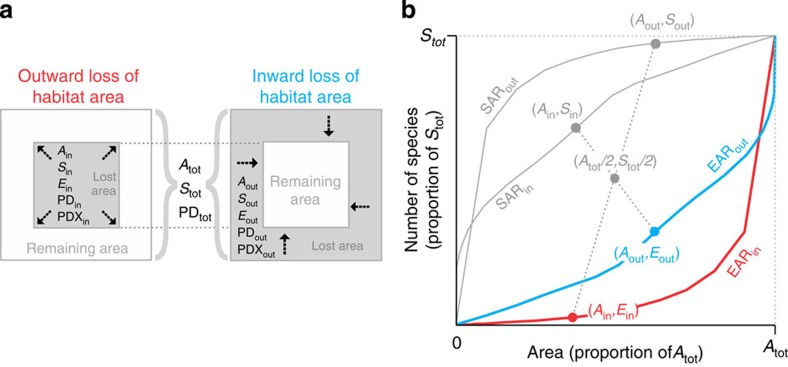
Schematic of inward and outward area loss and the corresponding endemics–area (EAR) and species–area (SAR) curves. The squares in (**a**) represent a hypothetical region with part of its habitable area lost. (**b**) Empirical curves for birds that inhabit 2,200 × 2,200 km^2^ placed in South America (region SA2) as an example. In both panels, *A*, *S* and *E* are area, number of species and number of extinct species in the inner (in) and outer (out) area, respectively, all expressed as proportions of total (tot) area and diversity.

We begin by showing the complementary nature of different ways to calculate the decline of species richness under area loss (Fig. 1), independent of any specific SAR model (for example, power-law as in refs 12, 18): Imagine a region of total area *A*_tot_ that hosts *S*_tot_ species, with a contiguous plot of area *A*_in_ (*A*_in_<*A*_tot_) somewhere within the region, with area outside the plot *A*_out_=*A*_tot_−*A*_in_. The number of species that live exclusively within (are endemic to) the plot (*E*_in_) is then given by *E*_in_=*S*_tot_−*S*_out_, where *S*_out_ is the total number of species (both endemic and non-endemic) that live in *A*_out_. With *S*_in_ defined as all species occurring in *A*_in_, it follows that *E*_out_=*S*_tot_−*S*_in_. When habitats in *A*_in_ are destroyed, we speak about *outward* loss (Fig. 1a, left), whereas *inward* loss happens when habitats in *A*_out_ are destroyed (Fig. 1a, right). The *E*_in_ and *E*_out_ scale with area according to their respective EAR_in_ and EAR_out_ relationships, whereas the *S*_in_ and *S*_out_ do so according to the SAR_in_ and SAR_out_ relationships (Fig. 1b). The EAR_in_ thus follows a point reflection symmetry with SAR_out_ and analogically EAR_out_ is symmetrical to SAR_in_ (ref. 13).

Assuming immediate extinction (that is, no extinction debt), relevant for extinction estimates are always the EAR curves, which we also call *extinction curves*. The number of extinct species can be calculated directly from the EAR for the lost area, or indirectly by rotating the SAR for the remaining area on its axis about a central point^14^,^26^ (Fig. 1b); the latter being analogous to the *backward method* of estimating extinctions. The backward approach was considered incorrect by He and Hubbell^12^, but recognized as valid by others^14^,^27^, with differences from the forward (that is, direct or EAR-based) method arising from differing geometries of area loss. Specifically, in the case of contiguous area loss, the forward (EAR-based) method represents area loss that goes outward from within, whereas the backward (SAR-based) method corresponds to inward loss starting from the edges of a region^14^.

### Theoretical predictions

**Figure 2:**
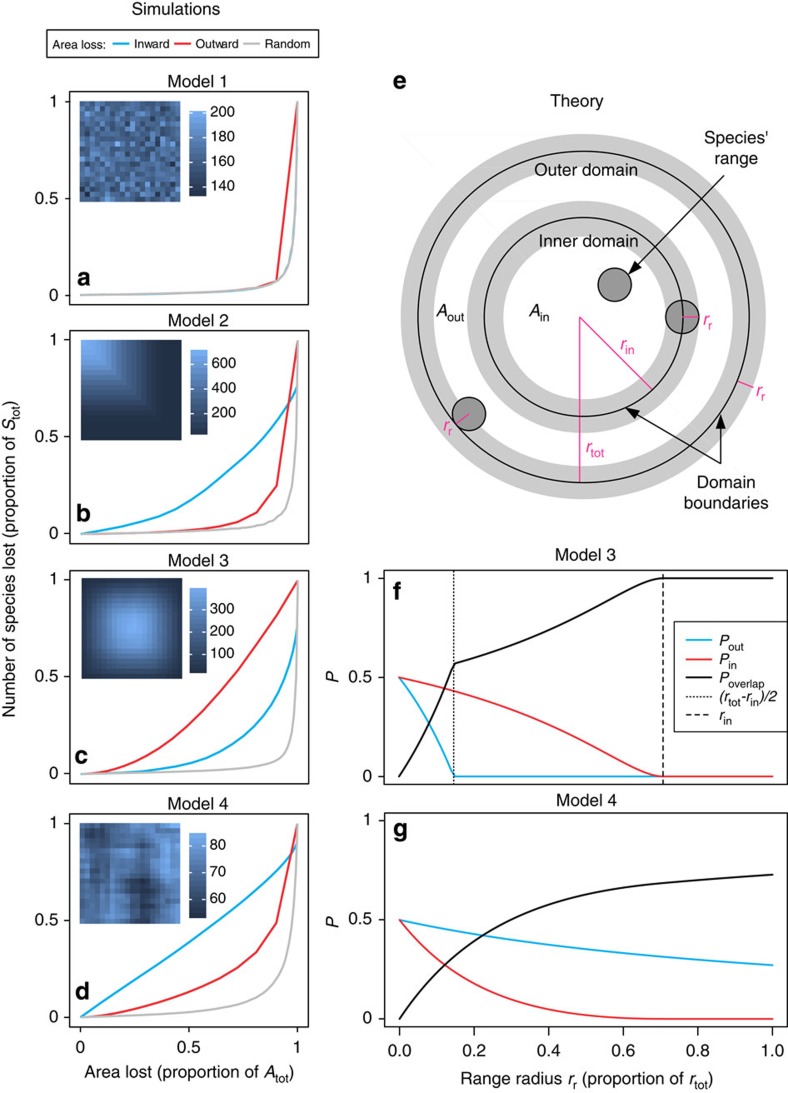
Expected differences between the inward and outward area loss based on theory and simulations. (**a**–**d**) Simulated extinction curves from four models of range placement and three scenarios of area loss. The insets show spatial patterns of species richness in a 20 × 20 cell domain, with lighter shades representing higher richness. The blue and red extinction curves are for inward and outward loss, respectively, the grey curves represent the scattered random loss scenario. (**e**–**g**) Theoretical demonstration of the probability that a randomly placed range falls exclusively within the inner (*P*_in_) or outer domain (*P*_out_), respectively, as a function of geographic range radius (*r*_r_). (**e**) Illustration of the terms used in the theoretical predictions: a contiguous circular range of radius *r*_r_ is placed into a circular region of radius *r*_tot_; the region of area *A*_tot_ is divided into the inner domain (with area *A*_in_ and radius *r*_in_) and outer domain of area *A*_out_. Grey areas refer to regions where it is impossible to locate centres of the range with radius *r*_r_ so that the range does not cross the boundary of some of the domains (that is, species cannot be endemic in one of the domains). Two models of random placement of contiguous ranges (Model 3 with mid-domain effect in **c**; Model 4 without mid-domain effect in **d**) give inverse relative positions of EAR_in_ and EAR_out_. The vertical axes in **f**–**g** refer to the probability that a range with given radius falls into the inner (red line) and outer (blue line) domain, or that it overlaps both domains (black line).

How severely diversity declines with area loss is reflected by steepness of EAR extinction curves, which in turn is determined by both the spatial arrangement of individual species ranges^26^ and of area loss. Using four distinct models of range placement (see Methods and Fig. 2 for details) and three scenarios of area loss in both analytical and simulation settings (Fig. 2), we make the following predictions:

Assuming *random placement of discontinuous scattered ranges* (Model 1; Fig. 2a), the inward and outward extinction curves should overlap; this coincidence of forward and backward curves for the randomly placed non-contiguous ranges stems from the same causes as the coincidence of complementary SAR, nested SAR and non-nested SAR for the random spatial distribution of individuals described by refs 12, 15.

Assuming *non-random concentration of contiguous ranges* in one part of the region (Model 2; Fig. 2b), trivial and predictable differences should emerge between the inward and outward EARs—when ranges are packed by the edges of the region (Fig. 2b) they will obviously be removed first by the inward area loss, and the opposite holds for ranges concentrated in the middle of the region. However, differences between EAR_in_ and EAR_out_ also emerge from models of random range placement (see below), so the difference is not just an issue of range concentration in particular regions.

Assuming *random placement of contiguous ranges* (Models 3 and 4, Fig. 2c,d and Supplementary Fig. 4), that is, when ranges within a region are distributed randomly and are convex and contiguous geometrical shapes, non-trivial differences between EAR_in_ and EAR_out_ emerge because of the different ways the randomness is modelled. For analytical arguments, we use circular ranges in a circular region (Fig. 2e–g; here we were inspired by ref. 28), whereas for simulation purposes (Fig. 2a–d) we use rectangular ranges on a rectangular grid.

Let us have a circular region of area *A*_tot_ and radius *r*_tot_, divided into an outer domain of area *A*_out_ and an inner domain of area *A*_in_ with radius *r*_in_ (Fig. 2e). Imagine that either *A*_in_ or *A*_out_ are completely destroyed, causing the loss of *E*_in_ or *E*_out_. Let us set *A*_in_=*A*_out_=*A*_tot_/2, and hence *r*_in_=*r*_tot_/
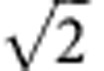
; in such case we are in the middle of the extinction curve (Fig. 1b), and any difference between *E*_in_ and *E*_out_ is due to reasons other than *A*_in_≠*A*_out_. Now when we try to place a circular range of radius *r*_r_ (Fig. 2e) at a *random* location within the region, we realize that there are actually several ways to model such random placement^26^. Here we consider two most distinct ones, and call them Models 3 and 4:

In Model *3* the whole range is placed randomly, so that its entire body must fall within the region boundary, which causes the well-known *mid-domain effect*^29^ (Fig. 2c and Supplementary Fig. 4a). Of key interest is the probability *P*_out_ that the range falls exclusively (entirely) within the outer domain, contributing to *E*_out_, and probability *P*_in_ that the range will fall exclusively within the inner domain, contributing to *E*_in_. These probabilities are (see Supplementary Note 2 for further details):


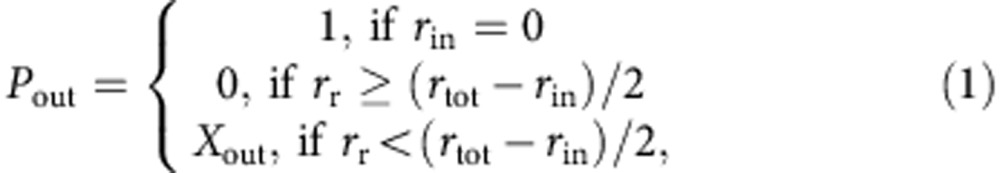


where


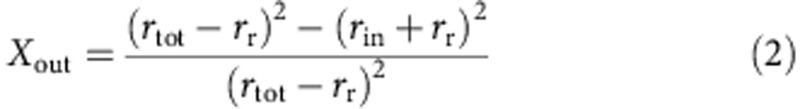


and


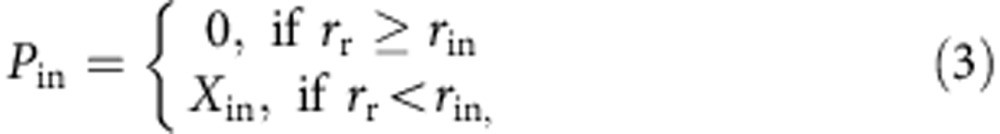


where


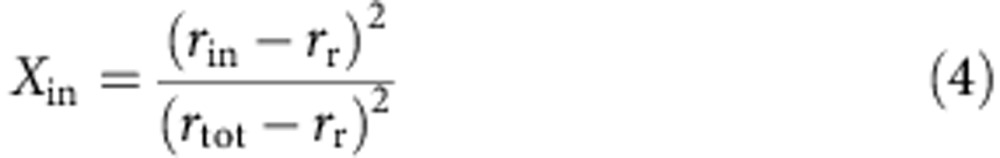


In Model 4, the range is allowed to overlap the region's outer boundary, and hence its area after the placement can be ‘cropped' by the boundary, effectively eliminating the mid-domain effect^26^ (Fig. 2d, Supplementary Figs 4a and 5). In this case, the probabilities are (see Supplementary Note 2 for further details):


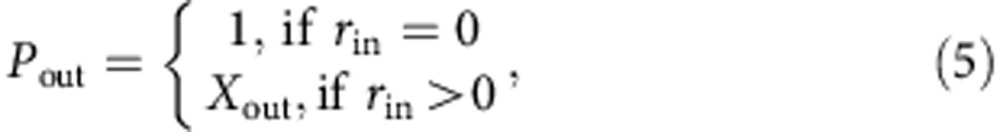


where


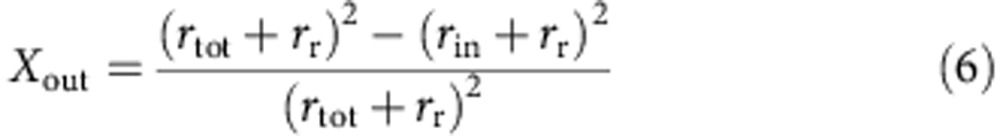


and


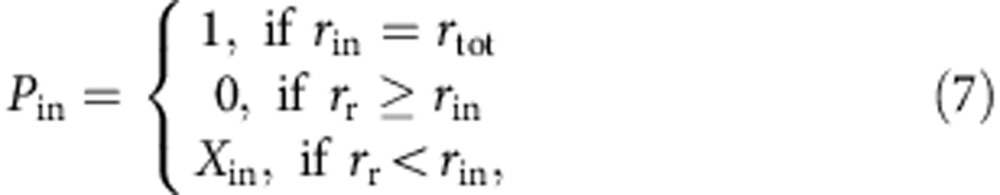


where


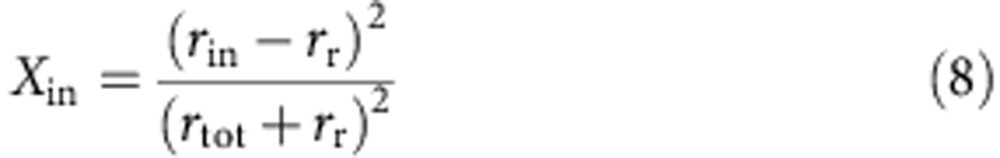


Here *r*_r_ is the radius of the potential circular range before its truncation by the region's boundary.

In Models 3 and 4, the probability that the range will overlap the boundary between the inner and outer domain is:*P*_overlap_=1−*P*_out_−*P*_in_ (9)

For *S*_tot_, species indexed by *i* it follows that 
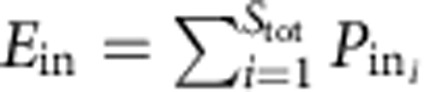
 and 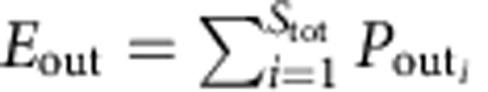. Figure 2f,g and Supplementary Figs 6 and 7 show that, for any *r*_r_, it always holds that *P*_in_≥*P*_out_ in Model 3, and hence, *E*_in_≥*E*_out_, whereas *P*_in_≤*P*_out_ in Model 4, and hence, *E*_in_≤*E*_out_. This holds irrespective of the shape of the range size frequency distribution, as the curves in Fig. 2f,g involve all possible range sizes (represented by *r*_r_).

Hence, we conclude that: when species ranges are contiguous and placed randomly and entirely within a region as in Model 3 (so that a ‘mid-domain effect'^26^ arises), and given *A*_in_=*A*_out_, loss of the inner area leads to a higher proportion of extinct diversity than loss of the outer area (Fig. 2c, f). When ranges are placed randomly, but are allowed to overlap the region boundary (that is, are ‘cropped' by it) as in Model 4, the loss of the outer area should be equally or more severe than loss of the inner area (Fig. 2d, g). Hence, different models of random range placement (that is, different null models) should lead to different relative positions of the EAR curves and consequently different extinction estimates. These analytical considerations are supported by our simulations (Fig. 2a–d).

### Declines of PD and FD

**Figure 3:**
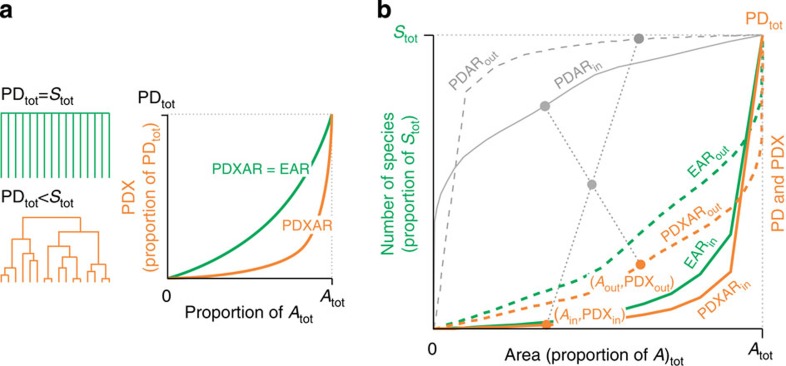
Relationship between EAR curves describing the loss of species (*E*) and PDXAR curves describing the loss of phylogenetic diversity (PDX) with area loss. (**a**) Illustrations of our expectation that two different phylogenetic trees lead to two distinct extinction curves (given the same spatial pattern of area loss) are shown. (**b**) The PDXAR versus EAR discrepancy in the context of the *inward* versus *outward* area loss, using empirical curves for South-American birds (region SA2) as an example. Note that the PDXAR and PDAR (that is, PD-area) curves follow the same point reflection symmetry as EAR and SAR, but the PD for the lost area cannot be used for the calculation of PD loss.

Compared with taxonomic richness, the loss of PD or FD (PDX or FDX, following the PDXAR and FDXAR curves; Fig. 3, Supplementary Fig. 2) is additionally affected by the structure of species' functional and evolutionary similarity with one another. In contrast to the loss of species richness, relative PDX or FDX, calculated as the total branch length that is lost from a dendrogram^24^,^30^, can only be calculated backwards by rotating the PD-area (PDAR) and FD-area (FDAR) curves of the non-destroyed area (Fig. 3 and Supplementary Fig. 2). Hence, the PDX cannot be calculated using the PD of endemic species alone, but requires information on the PD of the remaining species. Therefore, we need to know the complete phylogeny of all species in both the destroyed and the remaining area (Supplementary Fig. 2).

Lost species richness (*E*) is equivalent to PDX or FDX when all species are phylogenetically or functionally equivalent, that is, when the dendrogram representing their similarities is rake-shaped (Fig. 3a, green), and only such tree results in a proportional loss of PD (PDX) that is equal to that of *E*, that is, the PDXAR and EAR curves are identical. We predict that if a tree has ‘tippy' topology (Fig. 3a, orange), then a species that is randomly selected for extinction will, on average, represent lower proportion of the total branch lengths of the tree, compared with an extinction that occurs in a tree that has ‘stemmy' or rake-like topology (Fig. 3a, green). As a consequence, the initial loss of PD should be less pronounced than the loss of species richness, and the PDXAR curves should be below the EAR curves (Fig. 3b). In other words, any redundancy among species' functional or phylogenetic information, that is, increasing deviation from a rake-shape topology, will result in an initial loss of PD that is less pronounced than the loss of species richness. The same principles apply for the loss of FD (FDX) or other dendrogram-based metrics.

### Empirical extinction curves at large scales

**Figure 4:**
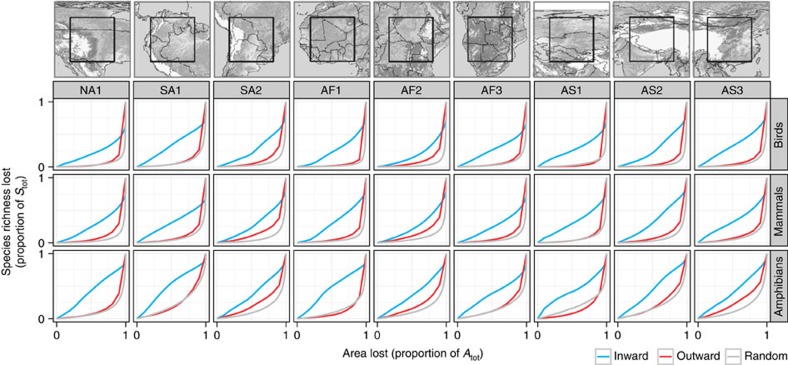
Loss of species richness (*E*) resulting from simulated area loss using empirical species distributions in nine regions and three taxa. Inward area loss (blue) always leads to higher proportional species loss than outward loss (red), and randomly scattered area loss (grey lines) consistently causes the lowest proportional loss. The curves are averages of multiple realizations of the habitat destruction in each of the nine regions and in each of the three vertebrate taxa (birds, mammals, amphibians). The maps at the top row show positions of the nine sampling regions. The shading in the maps indicates altitude.

**Figure 5:**
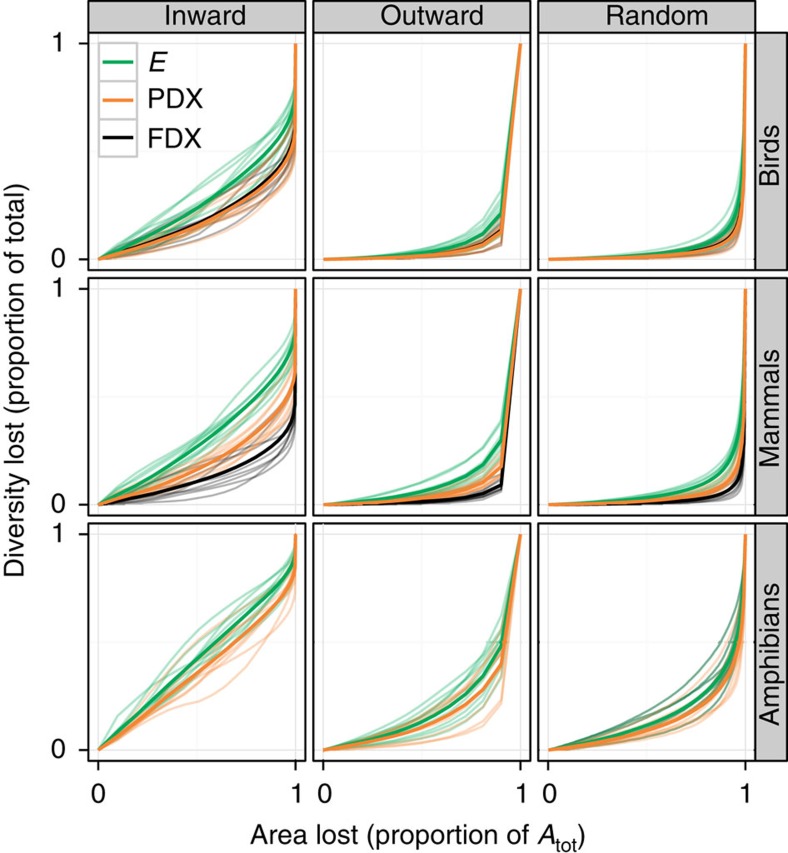
Loss of species richness (*E*) compared with the decrease of dendrogram-based measures of diversity (PDX and FDX) due to the simulated loss of habitable area in nine regions and three taxa. Thin transparent lines are realizations of the simulations in the nine regions, thick lines are their averages. Supplementary Fig. 3 provides detailed comparisons of the curves. Note that compared with mammals and birds the amphibian phylogeny has a weaker topological resolution and is based on a number of additional assumptions; see ref. 55 for details. We did not include the *E* versus FDX comparison for amphibians here as available data impeded comparability.

We find that for amphibians, birds and mammals in nine regions on four continents, simulated inward area loss leads to greater loss of species richness than outward area loss, and that the randomly scattered habitat loss leads to lowest loss of richness (Fig. 4). The proportion of species predicted to go extinct in a given area is generally highest for amphibians, which corresponds with this group's generally steeper SARs and EARs^26^. This is due to the relatively smaller ranges and higher endemicity of amphibians, which results in a predicted species loss that is almost proportional to the inward area loss. As expected, the initial loss of the PD and FD metrics PDX and FDX is always lower than the corresponding loss of species richness *E* (Fig. 5) for all taxa. This difference is particularly pronounced in mammals (Supplementary Fig. 3). In contrast, PD loss is relatively high in amphibians and also in birds in selected African and Asian regions (Supplementary Fig. 3).

### Predictors of regional extinction vulnerability

**Figure 6:**
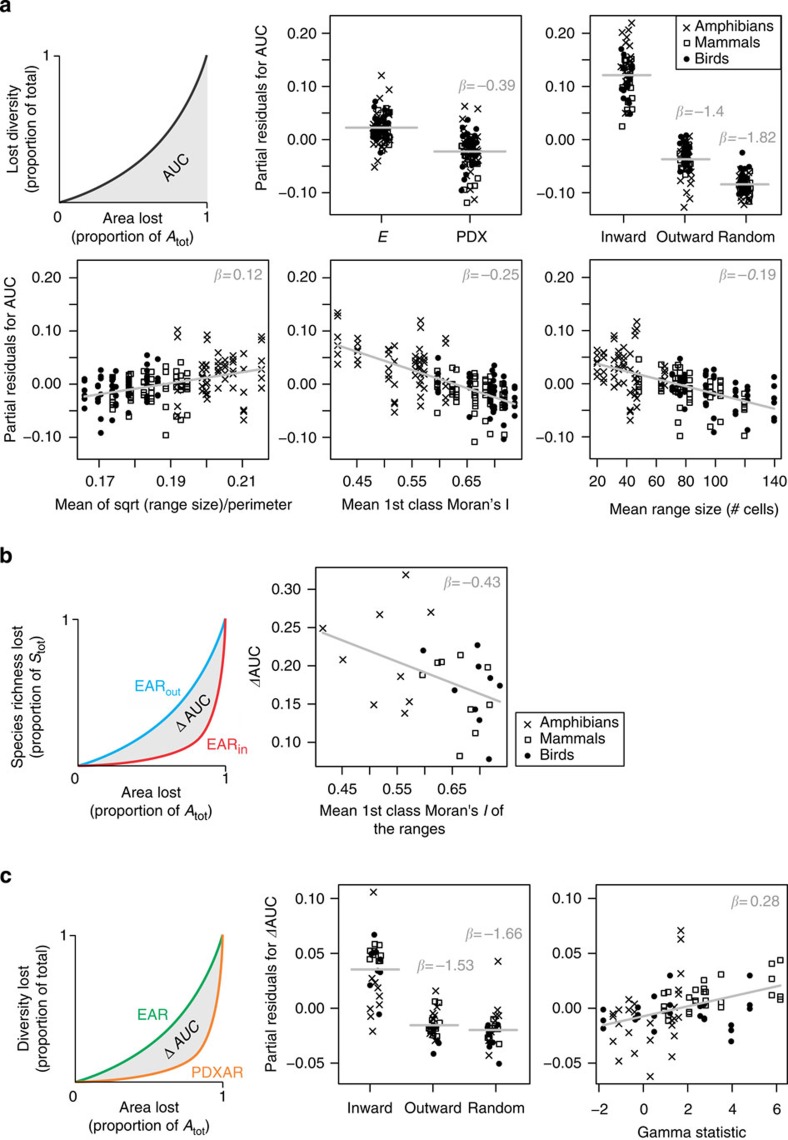
Statistical models explaining steepness of regional extinction curves and the discrepancy between different types of curves. (**a**) Predictors of regional extinction vulnerability as quantified by the AUC value, (**b**) the discrepancy (ΔAUC) between the inward and outward loss of habitat area and (**c**) the discrepancy between extinction curves for species richness (EAR) and phylogenetic diversity (PDXAR). Only the single best model, respectively, is shown (based on lowest AIC_c_ score), which includes different predictors in each of the three approaches (see Supplementary Tables 2–4 for details). Betas (*β*) are standardized coefficients. We do not provide *P*-values or standard errors because of a presumed but unknown degree of pseudo-replication (the same taxon over multiple regions, and vice versa). The Gamma statistic represents ‘stemminess' of a phylogenetic tree^61^. Each point stands for one taxon in one of the nine regions, and/or one measure of diversity where appropriate.

We use area under the extinction curve (AUC) as our measure of extinction vulnerability of a region, with steep curves, that is, high extinction vulnerability of a region, characterized by high AUC. The most important predictors of AUC were the Inward/Outward/Random geometry of the habitat loss (averaged (avg.) *β* of 0, −1.4 and −1.8, respectively; Supplementary Table 2) followed by the type of diversity considered (*E* or PDX; avg. *β*=−0.4; Supplementary Table 2), and three variables describing range geometry. Apart from the high extinction vulnerability for inward destruction and for species richness as a measure of diversity, we also found high vulnerability in taxa with relatively small mean range sizes (avg. *β*=−0.18), and with autocorrelated (avg. *β*=−0.26) and more compact (that is, relatively short perimeter; avg. *β*=−0.123) species' geographic ranges (Fig. 6a). In contrast, lower extinction vulnerability emerges for outward and randomly scattered destruction, for PD, and in regions and taxa with large, elongated and/or scattered ranges (Fig. 6a). This confirms our expectations about the role of range size^26^ and shape. Notably, the geometry of area loss (inward versus outward or randomly scattered) had a stronger effect than all other considered factors (Fig. 6a), including mean geographic range size.

### Predictors of discrepancy between inward and outward loss

Two predictors of the EAR_out_-EAR_in_ discrepancy (measured as ΔAUC) had particularly high absolute values of averaged beta coefficients and occurred in the two best models (Supplementary Table 3): the mean Moran's *I* of the ranges (avg. *β*=−0.43) and the mean range size (avg. *β*=−0.32). Specifically, the discrepancy was higher in regions and groups with smaller and less autocorrelated ranges. These two predictors were strongly collinear (Pearson's *ρ*=0.7, Supplementary Table 1) and hence we were unable to discriminate between them. Because of its slightly higher absolute value of *β* we report the mean Moran's *I* in Fig. 6b, but we stress that mean range size may play similarly important role as the autocorrelation of the ranges.

### Predictors of discrepancy between EAR and PDXAR

The most important predictors of the discrepancy (ΔAUC) between the loss of species richness (EAR) and loss of PD (PDXAR) were the factor describing the geometry of habitat loss (inward, outward or random with averaged *β* of 0, −1.53 and −1.66 respectively; Supplementary Table 4) and the Gamma statistic characterizing the tree (avg. *β*=0.35; Supplementary Table 4): phylogenetic trees characterized by higher concentration of branching events towards the tips (higher Gamma) lose their PD at a relatively slower rate. We report the model that contains these two predictors in Fig. 6c. Tree ‘stemminess' emerges as additional relevant predictor (avg. *β*=0.21; Supplementary Table 4), suggesting that the effect of tree topology on the EAR-PDXAR discrepancy is better captured by several tree summary statistics, rather than by the Gamma alone.

## Discussion

As inward versus outward extinction curves correspond to SAR-based (backward) versus EAR-based extinction estimates, our results falsify the statement by He and Hubbell^12^ that ‘Species–area relationships *always* overestimate extinction rates from habitat loss'. More precisely, our models show that the SAR-based backward method (equivalent to inward habitat loss^14^) can give higher estimates of diversity loss than the direct EAR-based method, but it can also give lower estimates, depending on the specific arrangement of species ranges in the region. This ambiguity emerges at large scales where species distributions are better described by contiguous blocks rather than by sets of individuals (as in ref. 12). The issue arises even under simple null expectations of random distribution of ranges, and it critically depends on whether the realized (observed) species distributions emerge as a result of truncation of potential distributions by physical barriers^29^ or whether some variant of mid-domain process generates the distributions^31^; our findings bring this long-standing debate from basic macroecology into the context of applied extinction science.

Our key empirical finding is that, at large geographic scales, the inward loss of habitats leads to more pronounced declines of species richness than when area is lost from within towards the edges. Our models indicate that this can happen for at least two reasons: (i) ranges may be non-randomly concentrated close to the edges for ecological reasons, for example, because of the presence of suitable habitats in those areas. (ii) Alternatively, the higher relative impact of inward area loss is expected in randomly distributed contiguous ranges, when the ranges are truncated or ‘cropped' by region boundary, and this truncation can happen for natural reasons, for example, coast truncating potential ranges of a terrestrial species^29^ (Supplementary Fig. 5b), but it can also be an artefact of the study design, for example, when the focal region is part of a larger region (Supplementary Fig. 5c), so that ranges along the edge of the focal region are only parts of larger ranges, overlapping the region boundary. This can easily happen when EAR curves are constructed for small-scale plots (as in ref. 12) that are arbitrarily delineated within a substantially larger region. In such a case, the discrepancy between the effect of inward and outward area loss is not particularly relevant for global species extinction, as the species that go extinct within the delineated area are mostly those that persist outside of it.

Exact quantification of the role of (i) and (ii) is beyond our scope here. Instead, we provide an inductive statistical model that predicts the shape of extinctions curves and their discrepancies by factors that are known to reflect scaling patterns of diversity^32^. We find that the inward–outward discrepancy is highest when ranges are small and have low spatial autocorrelation (contiguity), but these two aspects are correlated and difficult to separate. Intriguingly, the geometry of area loss (inward versus outward direction of loss) had a substantially stronger effect on the steepness of the extinction curves than mean geographic range size. Given the broadly recognized role of range size for extinction risk^33^,^34^, these findings are remarkable and point to the need to jointly account for both the range size and the spatial pattern of the lost habitable area.

Although the inward versus outward dichotomy represents useful extremes, real-world habitat destruction rarely happens in such a defined and contiguous form. Instead, often both inward habitat loss (for example, from larger-scale agricultural development or sea-level rise^35^,^36^,^37^) and outward area loss from localized centres (for example, sprawling cities or large-scale mining operations) may occur simultaneously. An extreme case of such simultaneous loss is captured by our random fragmentation scenario in which the SAR- and EAR- based extinction curves are equivalent. For this scenario, the magnitude of extinctions is always lower than in the contiguous scenarios (as also reported by refs 20, 21, 38), revealing the seriousness of the threat from increasing contiguous habitat transformations, as opposed to the more scattered alterations. Note, however, that this applies only to immediate biodiversity loss. The scattered area or habitat loss may actually lead to range fragmentation, which makes species more vulnerable to extinction in the future^16^,^17^. More realistic models that account for various biological phenomena such as coexistence mechanisms^16^, extinction debt^21^,^39^,^40^ or minimum area requirement^41^ could help narrow the often broad bounds on expected biodiversity loss provided by our extreme and simplified scenarios. We also see a promising new avenue in the emerging concept of countryside SAR^39^, which realistically assumes that the new habitats replacing the original area are still to some degree habitable. Unfortunately, some of the more realistic and complex models require relatively detailed and context-specific information^16^ and will thus be less generally applicable than the more tractable scenarios presented here.

Some specific demonstrations of how PD can scale with area (PDAR) are now available^42^,^43^. However, as we show, such PDAR curves provide no information about the sensitivity of PD to area loss in the focal region. Addressing this sensitivity requires: (i) the knowledge of the PDAR *outside* of the focal area and (ii) the point reflection symmetry of the PDAR and PDXAR curves. These are fundamental issues that require consideration in assessments and policy addressing PD decline under area loss. As predicted (and previously qualitatively argued for PDAR curves^42^), the discrepancy between the loss of species richness (described by EAR) and loss of PD (described by PDXAR) is determined by the shape of the underlying phylogenetic tree: the loss of PD (and other dendrogram-based metrics such as FD) is less pronounced when the extinct species have close relatives or functionally similar species in the region, that is, in a ‘stemmy' rather than ‘tippy' tree shape. For instance, although a single sunbird species is lost as habitable area disappears, other members of the clade would retain a diminishing core of the group's evolutionary and functional attributes, such as mixed nectar and insect feeding, until the extinction of the clade's final species.

We find that for terrestrial vertebrate groups and their analysed functional traits, FD suffers a generally less steep proportional loss than PD, highlighting that FD may often, and at least initially, be more readily retained compared with PD. However, unlike PDXAR, the specifics of the FDXAR curve and the generality of this finding will depend on the traits assessed^44^, their number and respective weighting, and the clustering algorithm (or FD metric), all of which can influence the topology of the functional dendrogram^45^,^46^. Here, the key insight pertains to qualitative comparison, that is, we can confidently claim that the initial loss of FD (and other dendrogram-based metrics) will always be less pronounced than the loss of species richness.

Although the theory on spatial scaling of biodiversity has seen considerable progress^26^,^47^,^48^,^49^, the scale-dependence of biodiversity loss is a complex and yet underdeveloped field, and further analyses into the associations that we uncovered are needed. In line with others^20^,^21^,^38^, we have shown that estimates of diversity loss based solely on area lost, and ignoring spatial shape (arrangement, direction) of the loss, can be misleading. The geometry of area loss is crucial, and must be accounted for whenever dealing with extinction predictions or management decisions. Although habitat transformation of small scattered areas within a region may be relatively benign until habitat fragmentation leads to large-scale disappearance of whole species ranges, the destruction of large contiguous blocks of habitat within a region may be fatal. Similarly, estimates of counts of species are agnostic to species' phylogenetic or functional distinctness or redundancy. Phylogenetic data, together with at least basic geographic distribution characteristics and additional trait information, is rapidly growing and should increasingly allow similar evaluations to ours for other clades and regions and enable a more inclusive conservation science that goes beyond vertebrates. As we demonstrate, such information can bound biodiversity loss expectations and can provide important baseline estimates of the multi-facetted ecological consequences of ongoing and future habitat loss.

## Methods

### Models of range placement

Here we describe four contrasting models of range placement that were used for our theoretical arguments (Fig. 2). The simulated realizations of Models 1–4 (Fig. 2a–d) use rectangular ranges placed on a rectangular grid, which is computationally convenient. The analytical reasoning based on Models 3 and 4 (Fig. 2e–g) assumes circular ranges placed into a circular region, which is analytically tractable^28^.

In Model 1, we assume random placement of scattered ranges. This model uses a region consisting of 20 × 20 grid cells of the same area, where each grid cell has a probability *P*_*i*_ of being occupied by an *i*-th species, and where *P*_*i*_ is constant across grid cells. The presence or absence of *i*-th species in each cell is then simulated as an outcome of Bernoulli process with probability *P*_*i*_. This model produces no spatial aggregation of ranges, and is similar to the random model of He and Hubbell^12^. When averaged across many realizations, this model gives identical EAR_in_ and EAR_out_ curves^12^. Note that He and Hubbell^12^ demonstrate this only implicitly and use different terminology; they also operate at fine scale using individuals as smallest spatial units, whereas here we consider rectangular grid.

In Model 2, we assume non-random placement of contiguous ranges. Real-world large-scale distributions are usually to some degree contiguous, and often aggregated. Model 2 takes this to the extreme by using only contiguous ranges and placing all of them into the upper-left corner of region consisting of 20 × 20 grid cells. As a consequence, when *A*_in_=*A*_out_ the difference between *E*_in_ and *E*_out_ will simply reflect the number of ranges forced to fall completely into *A*_out_. This mimics, for example, the presence of strong environmental gradients or barriers constraining (truncating) species distributions, and in such a case, the difference between EAR_in_ and EAR_out_ is trivial.

Models 3 and 4 use random placement of contiguous ranges (Supplementary Fig. 4). Model 3 places each contiguous range entirely and randomly within the region, causing highest richness in the centre (mid-domain effect). Model 4 also places ranges randomly, but allows them to overlap domain edges, effectively eliminating the mid-domain effect (see ref. 26 for details of this algorithm).

### Simulations from models of range placement

The simulations use square ranges placed on an artificial rectangular grid (region) of 20 × 20 grid cells (Fig. 2a–d; see refs 16, 26 for similar approach). In each simulation run, we place 711 ranges with sizes drawn from empirical range-size frequency distribution observed in the species of birds that inhabit 2,200 × 2,200 km^2^ placed in Western Africa (region AF1 in Fig. 4). In each run, we calculate mean extinction curves of the inward and outward scenario (EAR_in_ and EAR_out_), as well as scattered random destruction scenarios in which grid cells are lost stepwise, one by one, and with the same probability at each step. Each simulation run was repeated 100 times, and the resulting 100 extinction curves were averaged to produce Fig. 2a–d. The same algorithm was applied to produce the empirical extinction curves (see below).

### Vertebrate distributional data

We used expert-drawn range maps for terrestrial amphibians, birds and mammals in all analyses. The range-maps were based on the IUCN assessment (http://www.iucnredlist.org/) for mammals^50^ and amphibians^51^. Distributions for birds were compiled from the best available sources for a given geographical region or taxonomic group^1^. See also ref. 52 for more details on the data.

### Grid system and study regions

We used an equal area grid originally derived in cylindrical equal-area projection with∼1° size at the equator and a constant grid cell area *A*_cell_ of ∼110 × 110 km^2^. We selected nine square regions (hereafter *the regions*, Fig. 4 and Supplementary Fig. 1), each of them having a total area (*A*_tot_) of exactly 20 × 20 grid cells. The regions were selected so that they lie completely within major continental landmasses and each grid cell within the region contains at least some land. The regions were also selected to cover broad variety of terrestrial biomes, altitudes and latitudes. The nearly identical geometry (note that regions in higher latitudes are slightly elongated relative to the low latitudes) of the regions enabled us to control for the potentially confounding effects of area and shape, and the extinction curves are thus comparable among the nine regions. We note that because of their large size, the study regions are bound to contain distinct environmental gradients, and species ranges inside the regions may aggregate along such gradients, similar to the situation in Model 2. This has the potential to enhance the inward–outward discrepancy of the EAR curves.

### Extinction curves: species richness (E)

We explored three scenarios of habitable area loss: inward, outward and scattered random. We adopted a strictly nested sampling design^26^ to explore the effects of *inward* and *outward scenario* (Fig. 1). We considered sampling windows of sides *l* and area *A*_in_=*A*_cell_ × *l*^2^, where 
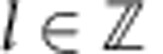
 and 1≤*l*≤20. We started with the smallest sampling window (*l*=1) and with the largest area outside of the sampling window (*A*_out_=*A*_tot_−*A*_in_). We moved the sampling window continually across the 20 × 20 regional grid, and for each position, we recorded number of species with ranges exclusively within (*E*_in_) and number of species with ranges exclusively outside of (*E*_out_) the sampling window. We calculated mean 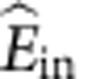 and 
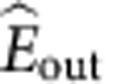
 by averaging *E*_in_ and *E*_out_ values across all of the possible positions of the sampling window. We then enlarged the side of the sampling window to *l*=2 and we repeated the procedure described above. We continued enlarging the window until *l* reached 20 and *A*_in_=*A*_tot_, calculating mean 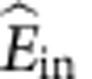 and 
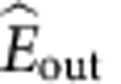
 for each of the window areas. We then plotted the proportions 
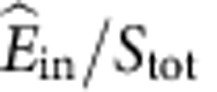
 against *A*_in_/*A*_tot_ (these are the red EAR_in_ curves in Figs 1 and 4 representing the *outward* destruction) and 
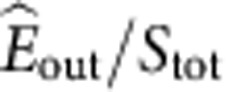
against *A*_out_/*A*_tot_ (these are the blue EAR_out_ curves in Figs 1 and 4 that represent the *inward* destruction).

In the *scattered random destruction scenario*, we selected the grid cells for the sampling one by one. The area within the set of selected cells was *A*_in_, and the richness of species endemic to that area was *E*_in_. In each step, each grid cell within the remaining area (*A*_tot_−*A*_in_) had the same probability of being selected for the destruction. We repeated this procedure 400 times and averaged the resulting extinction curves to get EAR_in_ and EAR_out_.

### Extinction curves: PD

We used the most recent and dated phylogenies on the three vertebrate taxa. For birds, we chose the first tree in the posterior set (distribution) of trees in Jetz *et al*.^53^ (see also Jetz *et al*.^23^ for discussion of tree dating and robustness). We also selected one mammal super-tree from Kuhn *et al*.^54^. For amphibians, we used the super-tree from Isaac *et al*.^55^. We used the term PD for the sum of the branch lengths of a phylogenetic tree (or a sub-tree, see below)^24^. In one extreme case (the rake-shaped phylogeny), PD is equivalent to species richness (Fig. 3). Let us define several terms (see also Fig. 3 and Supplementary Fig. 2): Let PD_tot_ be the total PD of a region, PD_in_ and PD_out_ are PDs of all of the species occurring inside and outside of the sampling window, respectively, PDE_in_ and PDE_out_ are PDs of species that live exclusively (are endemic to) inside and outside of the window, and PDX_in_ and PDX_out_ are the PD lost due to area loss. We note that PDX_in_ does not always equal PDE_in_ as we demonstrate in Supplementary Fig. 2b—PDX_in_ is calculated using PD_out_, whereas calculation of PDE_in_ does not require PD_out_. In fact, PDX_in_=PD_tot_−PD_out_ (Supplementary Fig. 2), and the same principle applies for PDX_out_. For our purpose, we calculated 
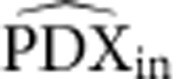
 and 
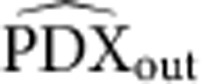
, which is the PDX_in_ and PDX_out_ averaged over all of the positions of the sampling window of a given area. We plotted the proportions 
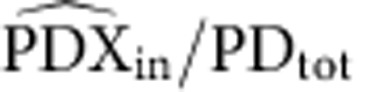
 against *A*_in_/*A*_tot_ (the solid PDXAR_in_ curve in Fig. 3, representing *outward* habitat loss) and 
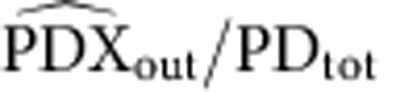
against *A*_out_/*A*_tot_ (the dashed PDXAR_out_ curve in Fig. 3, representing the *inward* habitat loss).

### Extinction curves: FD

We used species-level trait databases of all birds and mammals^56^ to calculate dendrogram-based FD metric for all grid cell assemblages^57^. Specifically, we used Gower distance to calculate species pairwise dissimilarity, weighting each of five functional trait categories (diet, body size, activity time and two measures of foraging niche) equally. For detailed description of the traits, see Wilman *et al*.^56^, Additional Information and Supplementary Methods. The species were then clustered by an UPGMA algorithm^58^ to obtain a functional dendrogram of each taxon in each region, an approach that in simulation studies has been shown to provide strong representation of original dissimilarities^59^. To calculate the proportional loss of FD (FDX), we applied exactly the same procedure as described above for PD, but using the functional dendrograms instead of the phylogenetic trees. We did not have a comparably comprehensive species-level trait database for amphibians, so we did not construct the FDX curves for this taxon.

### Representing regional extinction vulnerability by AUC

We use the AUC (or simply integral) to represent the abovementioned variation of extinction curves (EAR, PDXAR and FDXAR), with steep curves, that is, high extinction vulnerability of a region, characterized by high AUC.

### Regional predictors

For each of the three major taxa in each of the nine regions, we put together nine predictors (see Supplementary Methods for technical details): (i) *Gamma statistic.* Tree ‘stemminess' represented by Pybus and Harvey's^60^ gamma statistic (*γ*) characterizes the distribution of branching events within the tree. Trees with *γ*<0 have relatively longer inter-nodal distances towards the tips of the phylogeny (‘tippy' trees), whereas trees with *γ*>0 have relatively longer inter-nodal distances towards the root of the phylogeny (‘stemmy' trees). (ii) *Stemminess.* This is an alternative measure of how ‘tippy' (or ‘stemmy') a tree is in a given region. It is calculated as *L*_actual_/*L*_max_, where *L*_actual_ is the sum of all branch lengths in the phylogeny and *L*_max_ is the distance from the tips to the root of the tree multiplied by the total number of tips. (iii) *Colless' index of imbalance*^61^, which measures the branching symmetry of the phylogenetic tree of a given taxon in a given region. (iv) *Mean range size*, which is the arithmetic mean (calculated across all species) of the total number of grid cells in a given region in which a species was ‘detected' according to the expert-drawn range map. (v) *Mean of sqrt(range size)/perimeter.* By perimeter we mean the total number of grid-cell sides that form edges of a gridded area occupied by a species in a given region. We calculated the arithmetic mean of the sqrt(range size)/perimeter over all species of a given taxon that live in the region. (vi) *Mean Moran's I of the ranges.* For each species in each region, we measured the autocorrelation of the 1 (occupied) and 0 (unoccupied) values by global Moran's *I*^58^, which is a measure of contiguity of the ranges, and we took the arithmetic mean (across all species) of the values. (vii) *Total richness* (or *S*_tot_) of given taxon in given region. (viii) *Richness gradient.* We created fine-grain (using 1° cells) map of species richness for each taxon in each region. We then calculated an ordinary least squares linear regression of the richness in the cells against latitude and longitude of the cells, and their interaction. *R*^*2*^ of this regression is our index of richness gradient. We interpret it as the magnitude of the large-scale spatial autocorrelation of species richness; it also measures how clumped are species ranges towards the edge of the region. (ix) *Moran's I of richness*, which measured the global first-distance class autocorrelation of cell-specific values of species richness. In contrast to the previous measure, the Moran's *I* of richness captures short-distance autocorrelation.

Predictors i–iii capture various aspects of departure of phylogenetic trees from the rake-shaped topology, which we hypothesized to be responsible for the PDXAR-EAR discrepancy (Fig. 3 and Supplementary Fig. 2). Predictors iv–vi describe some basic geometrical properties of species geographic ranges in the regions such as range shape, contiguity and complexity of the range edge, which have potential links to scaling patterns of biodiversity^26^,^32^. Predictor vii represents the size of the species pool (regional diversity), which was also suggested to affect beta diversity^62^ and the associated scaling of species richness. Predictors vii and ix describe autocorrelation structure of gradients of species richness, which we expect to broadly correspond with aggregation of species ranges within the domain, as illustrated by our simulations in the inset maps in Fig. 2a–d.

### Models explaining AUC and ΔAUC by the regional predictors

We built three sets of statistical models: (i) Models that explain AUC of all of the EAR and PDXAR curves (162 data points). (ii) Models that explain ΔAUC of all pairs of EAR_in_ and EAR_out_ (27 data points). (iii) Models that explain ΔAUC of all pairs of EAR and PDXAR curves (81 data points). By ‘data points', we mean unique combinations of taxa, regions and types of extinction curves. All models used the predictors described above as predictors and the taxon- and region-specific AUC or ΔAUC as a response. The specific predictors used in each of the three sets of models are listed in Supplementary Tables 2–4. For all models, we used ordinary least squares regression (normal error distribution). We first fitted models with all combinations of predictors (no interactions or nonlinear terms) and we ranked the models by their AIC_c_ (Akaike Information Criterion with small sample size correction). For all predictors, we also calculated standardized regression coefficients (*β*) by rescaling each predictor to zero mean and variance of 1, and we averaged the betas over all of the models (Supplementary Tables 2–4).

All result from modelling, model selection and model averaging are summarized in Supplementary Tables 2–4. Models presented in Fig. 6 were selected among the models given in Supplementary Tables 2–4. When selecting these models we had in mind (i) their interpretability, (ii) out-of-sample predictive performance measured by AIC_c_, (iii) number of predictors, (iv) collinearity between the predictors (Supplementary Table 1) and (v) how well they corresponded with the *averaged* standardized coefficients (*β*) presented in the second line of Supplementary Tables 2–4. Finally, we note that our 27 combinations of taxa and regions used for the modelling give a relatively small sample size and are not independent realizations of a random process. This makes it problematic to calculate likelihoods and limits the interpretation of AIC_c_ values and model rankings (Supplementary Tables 2–4). Consequently, we do not report *P*-values or standard errors.

We note that the standard, published procedures for dendrogram-based FD calculations are affected by the number, weighting and types of traits assessed^44^, and the clustering algorithm^40^,^41^. These differences may impact the exact shape of FDXAR curves. We therefore do not analyse these curves or FDXAR and PDXAR differences in detail and instead focus on the qualitative comparison between FDXAR and EAR curves, which is not affected by these methodological effects.

All of the distributional data, phylogenies, functional dendrograms and shapefiles used for the analyses described above are also provided (see Additional Information). Further technical details are in Supplementary Methods.

## Additional information

**How to cite this article:** Keil, P. *et al*. On the decline of biodiversity due to area loss. *Nat. Commun.* 6:8837 doi: 10.1038/ncomms9837 (2015).

## Supplementary Material

Supplementary Information Supplementary Figures 1-7, Supplementary Tables 1-4, Supplementary Notes 1-2, Supplementary Methods and Supplementary References
