# Supplementary material for: On the decline of biodiversity due to area loss
Source: Nat Commun. 2015 Nov 17;6:8837. doi: 10.1038/ncomms9837 (PMC4660053; doi:10.1038/ncomms9837)
Supplement: Supplementary Information — Supplementary Figures 1-7, Supplementary Tables 1-4, Supplementary Notes 1-2, Supplementary Methods and Supplementary References [file ncomms9837-s1.pdf]

Supplementary Figures

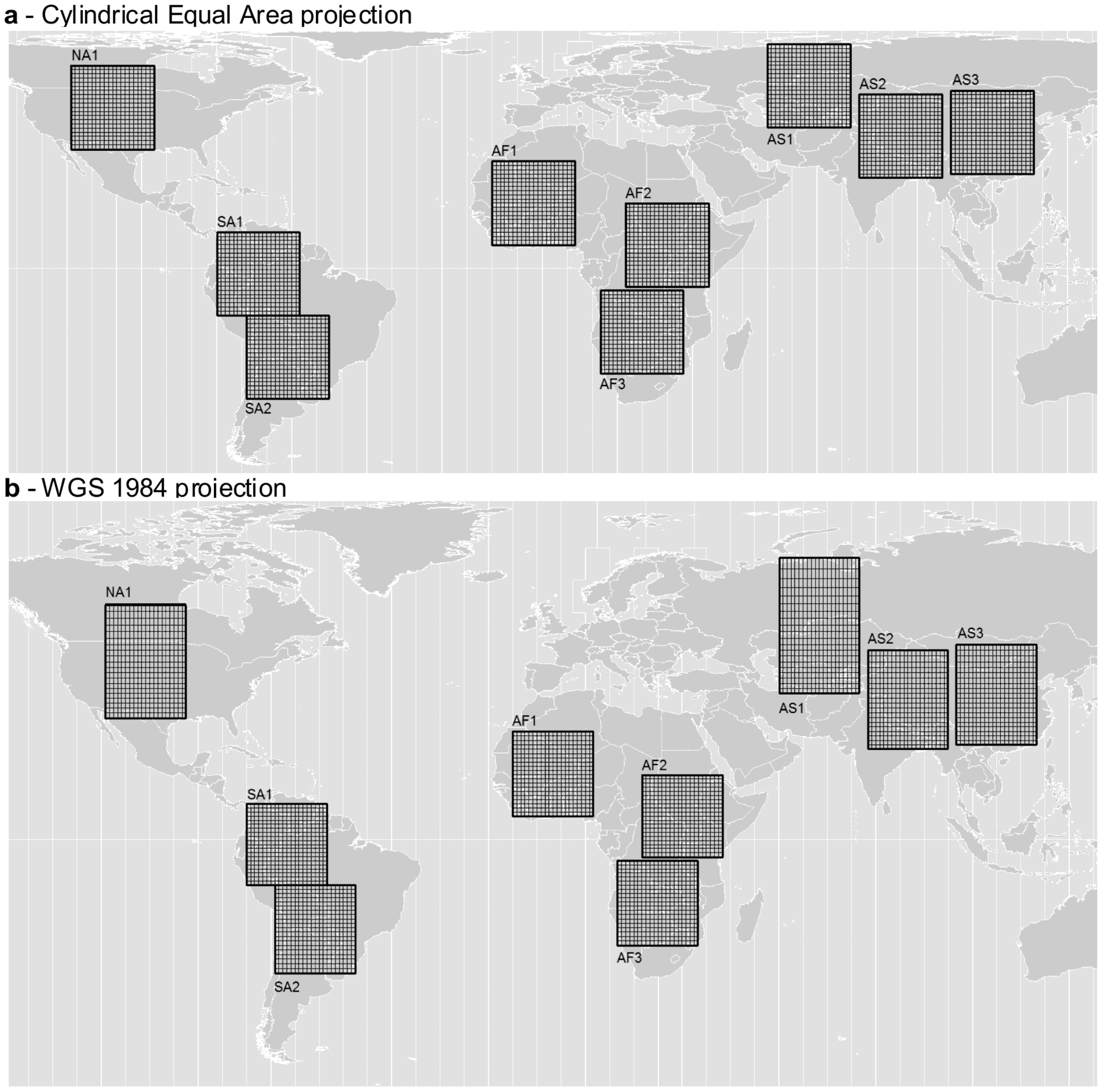

Supplementary Figure 1 | Locations of the nine sampling regions in the context of the whole World. Each region consists of 20 × 20 grid cells. Each grid cell has an area of approximately 110 × 110 km^2. In order to get grid cells of equal area as in (**A**) the grid cells in higher latitudes are elongated, as seen in (**B**).

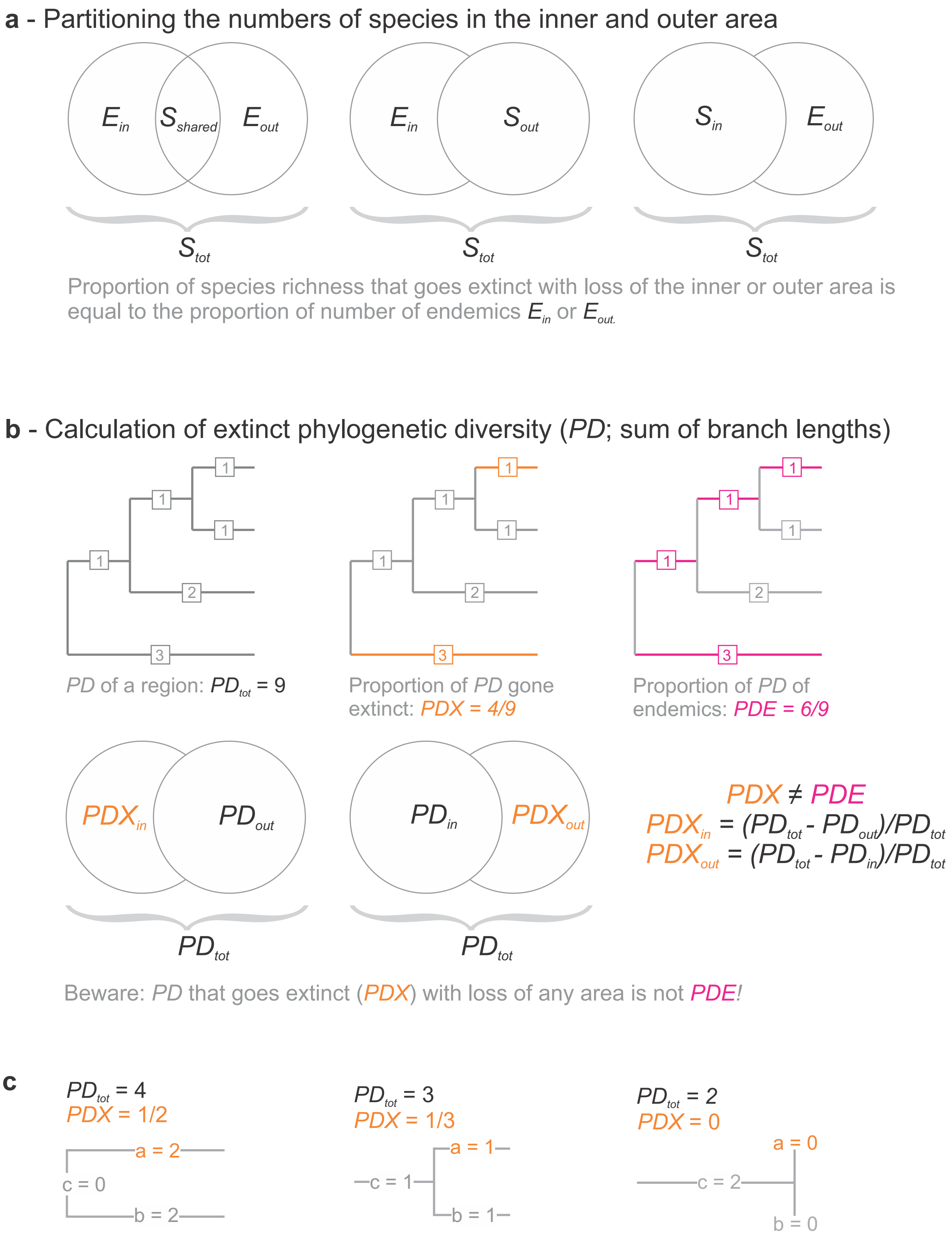

Supplementary Figure 2 | Scheme showing which components of species richness and phylogenetic diversity are lost with the loss of inner or outer habitable area. (**a**) The proportion of species richness that goes extinct is equal to the proportion of endemics (E) in the area lost. (**b**) The proportion of phylogenetic diversity that goes extinct (PDX) is not equal to the proportion of phylogenetic diversity of endemics in the destroyed area (PDE). (**c**) A simple illustration of how extinction of a species with a closely related survivor leads to lower PDX than extinction of a species with distantly related survivor. The second main point of our study (i.e. $PDXAR$ curves lay below the EAR curves) is based on this principle, which we also explain in the main text.

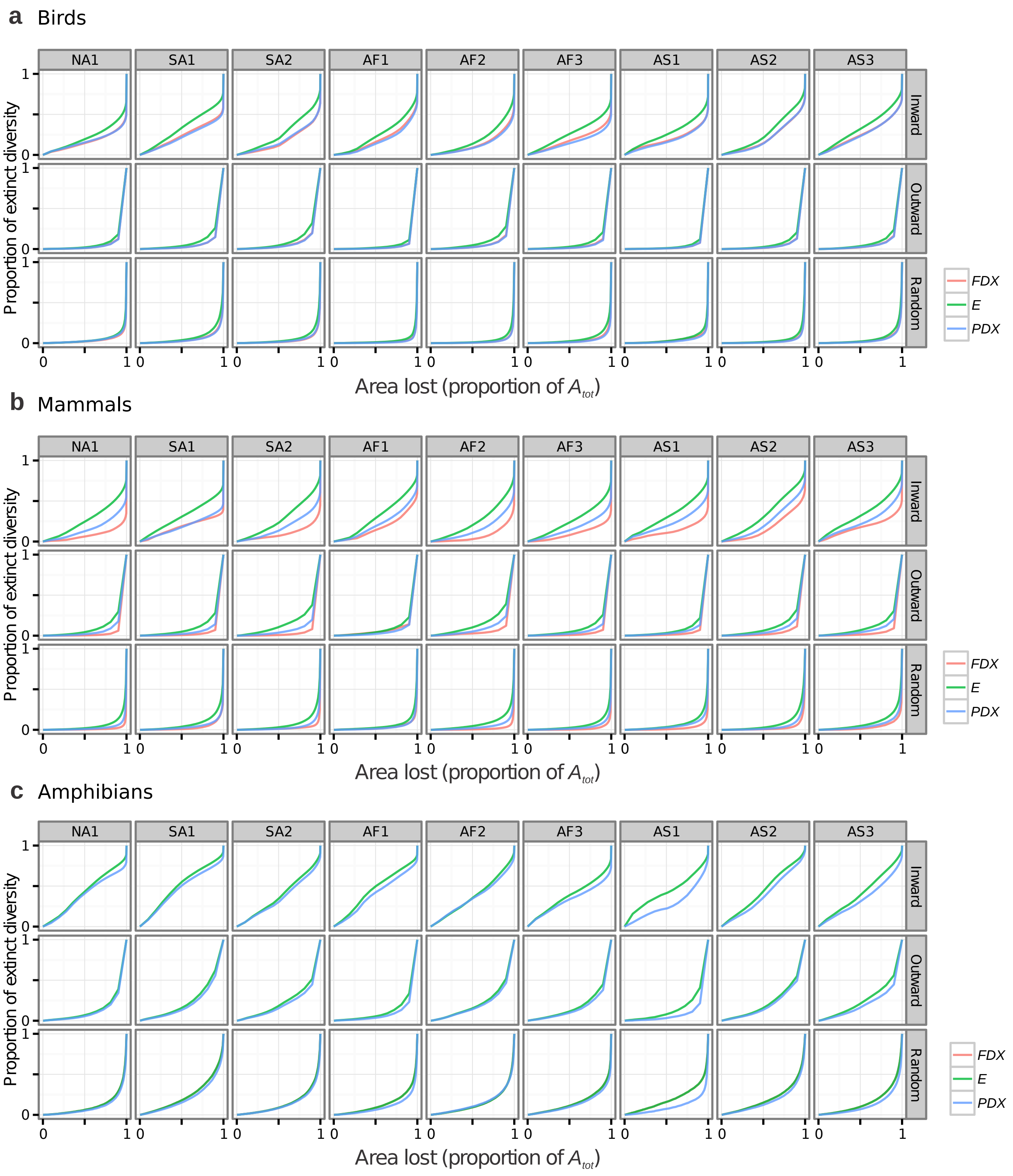

Supplementary Figure 3 | All of the extinction curves (EAR, PDX and FDX) for the three vertebrate taxa. Panel (**a**) shows birds, panel (**b**) mammals, and panel (**c**) amphibians.

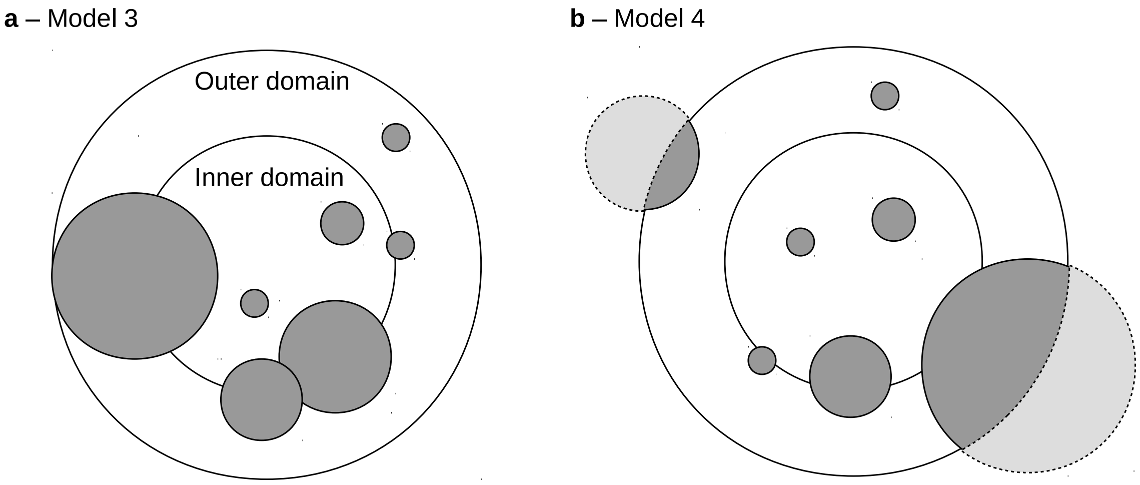

Supplementary Figure 4 | Schematic comparing range placement in Model 3 and Model 4. In Model 3 the ranges (dark grey circles) are placed randomly, but must never overlap the outer domain's outer boundary. In contrast, Model 4 allows the ranges to overlap the outer boundary, and the part of the range that falls outside of the boundary (light grey) is lost. As a result, realized frequency distribution of range sizes within the whole region can be different from the frequency distribution of the potential circular ranges.

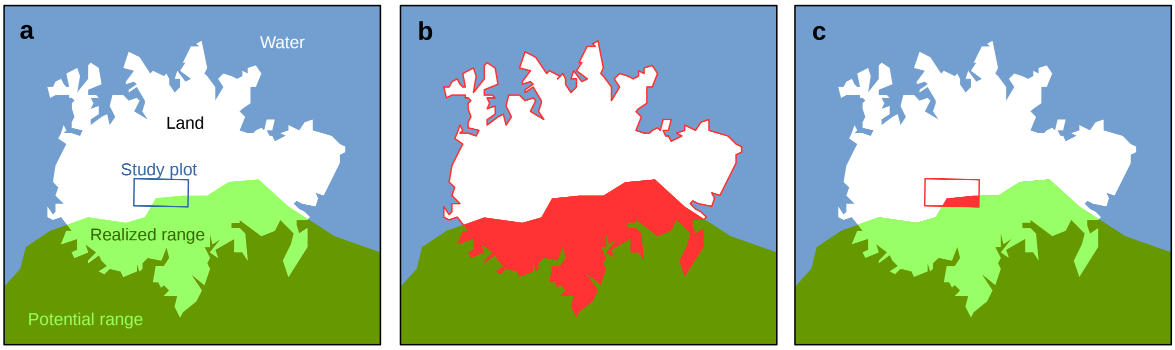

Supplementary Figure 5 | Relevance of Model 4 in real world. Panel (**a**) shows an island with an arbitrarily delimited study plot (the map is based on Barro Colorado Island in Panama). There is a hypothetical terrestrial species living in the southern part of the island (light green area); in the past there used to be no water and the species also used to live south from the island (dark green area), and it will be able to re-occupy the potential range if the water disappears. Panel (**b**) shows how the island coast truncates the potential range, while in panel (**c**) the artificial plot boundary truncates the realized range. When estimating extinction, one may be interested in extinction of the species only within the plot (**c**), within the whole island (**b**), or within the whole world. In any of these cases the species range, realized or potential, can be truncated by natural or artificial barriers, making Model 4 a useful null model of range placement.

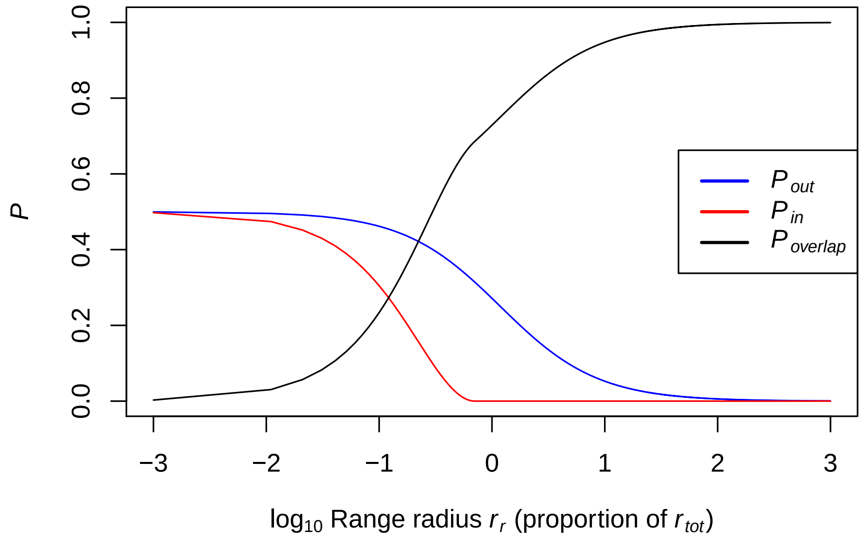

Supplementary Figure 6 | Extended version of Figure 2g. Here we show that, in Model 4, $P_{out} \geq P_{in}$ even when $r_r > r_{tot}$. Note that, in contrast to Figure 2g, the x-axis here has $\log_{10}$ scale; hence, valueg of -2 means that r_r is $100\times$ smaller than r_{tot}, 0 means that $r_r = r_{tot}$, and value of 2 means that r_r is $100\times$ larger than r_{tot}.

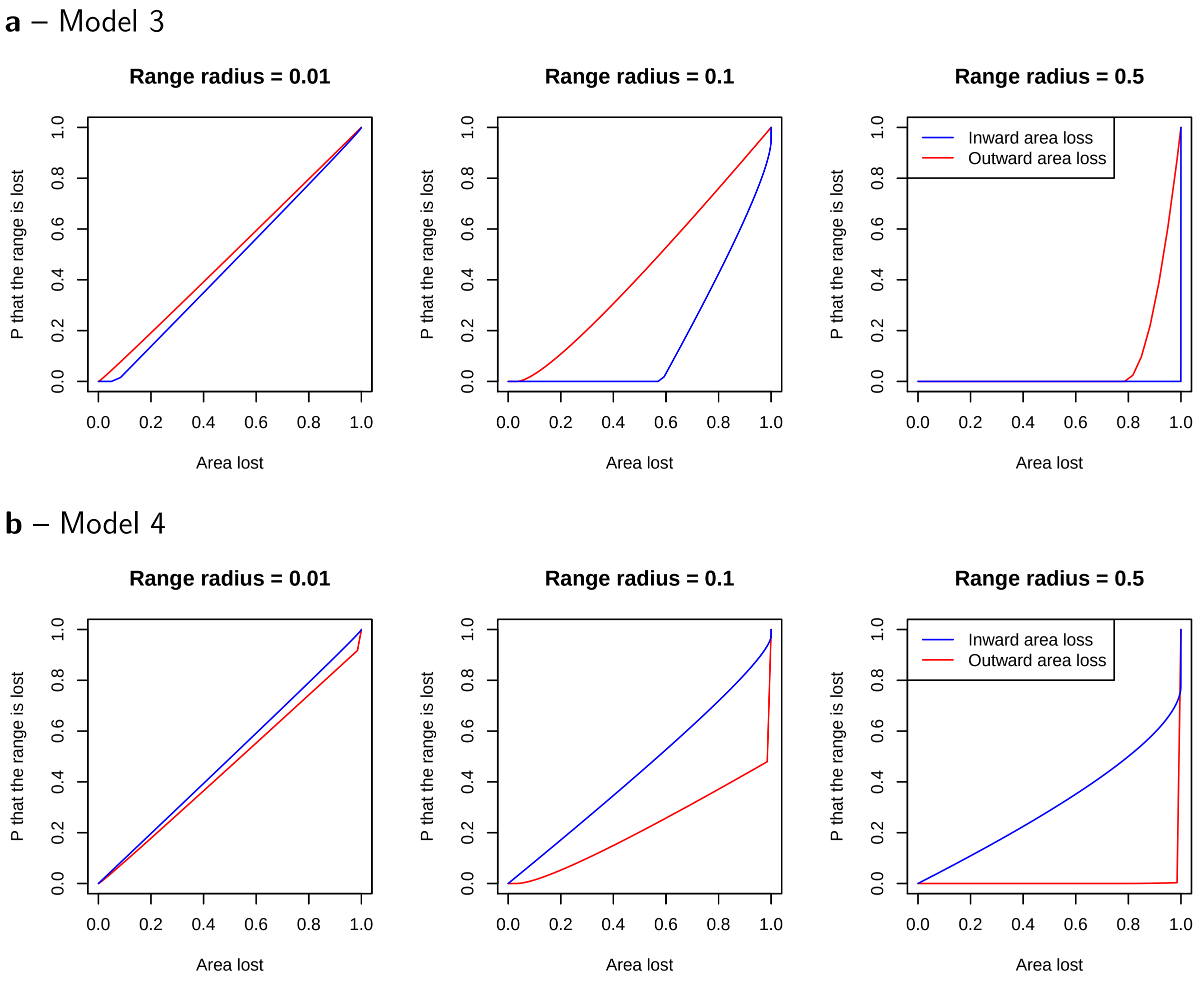

Supplementary Figure 7 | Probability that a single species' range placed according to Model 3 or model 4 will be lost with the loss of habitable area. We used equations 1, 3, 6 and 8 (Supplementary Note 2) to construct the curves for three values of range radius r_r: 0.01, 0.1 and 0.5. The total area of the region is 1.

Supplementary Tables

Supplementary Table 1 | Pearson's correlations of the regional characteristics that were used to predict AUC and ΔAUC (see the Methods for details). Cells marked by grey fill contain values higher than 0.5 or lower than -0.5 (an arbitrary threshold).

	Gamma statistic	Stemminess	Colless	Mean range size	Mean of sqrt(range size)/perimeter	Mean Moran's I of the ranges	Total richness	Richness gradient
Gamma statistic	1							
Stemminess	-0.35	1						
Colless	-0.14	-0.37	1					
Mean range size	0.38	0.01	0.24	1				
Mean of sqrt(range size)/perimeter	-0.14	-0.09	-0.35	-0.6	1			
Mean Moran's I of the ranges	0.18	0.12	0.12	0.7	-0.76	1		
Total richness	0.24	-0.29	0.78	0.38	-0.53	0.27	1	
Richness gradient	-0.32	0	-0.13	-0.28	0	0.16	-0.28	1
Moran's I of richness	-0.49	0.28	-0.12	-0.36	0.01	0.14	-0.34	0.75

Supplementary Table 2 | Forty models with the lowest AIC_c values (out of 256 models in total) explaining the AUC of all EAR and $PDXAR$ curves. Each model is one line of the table. Predictors used in each model are marked by '+'. The models are ranked by their AIC_c (Akaike Information Criterion corrected for small sample sizes). The second line of the table gives standardized regression coefficients (β) for a given predictor, averaged over all of the 256 models. Green filling marks the model that is illustrated in Fig. 6a of the main text.

	Inward/ Outward/ Random	E/PDX	Mean Moran's I of the ranges	Mean range size	Mean of sqrt(range size)/perimeter	Moran's I of richness	Richness gradient	Total richness	# of predictors	*AICc*	*ΔAICc*	*AICc* weight
Averaged standardized coefficient (β)	0/-1.4/-1.8	0/-0.4	-0.26	-0.18	0.123	0.011	0.026	0.009				
Fig. 6A	+	+	+	+	+				5	-642.434	0	0.361
	+	+	+	+	+		+		6	-641.058	1.376	0.181
	+	+	+	+	+	+			6	-640.461	1.973	0.134
	+	+	+	+	+			+	6	-640.262	2.173	0.122
	+	+	+	+	+		+	+	7	-638.993	3.441	0.065
	+	+	+	+	+	+	+		7	-638.788	3.646	0.058
	+	+	+	+	+	+		+	7	-638.363	4.072	0.047
	+	+	+	+	+	+	+	+	8	-636.691	5.743	0.02
	+	+	+	+				+	6	-632.451	9.983	0.002
	+	+	+	+					4	-632.361	10.073	0.002
	+	+	+	+			+		5	-631.97	10.464	0.002
	+	+	+	+			+	+	6	-631.269	11.165	0.001
	+	+	+	+		+			5	-631.074	11.36	0.001
	+	+	+	+		+		+	6	-630.505	11.929	0.001
	+	+	+	+		+	+		6	-629.758	12.676	0.001
	+	+	+	+		+	+	+	7	-629.001	13.433	0
	+	+	+		+	+			5	-624.172	18.262	0
	+	+	+		+	+	+		6	-623.487	18.948	0
	+	+	+		+		+		5	-622.022	20.412	0
	+	+	+		+	+		+	6	-622.02	20.414	0
	+	+	+		+	+	+	+	7	-621.347	21.088	0
	+	+	+		+		+	+	6	-619.783	22.651	0
	+	+	+			+			4	-615.563	26.871	0
	+	+	+			+	+		5	-615.364	27.7	0
	+	+	+			+		+	5	-615.131	27.303	0
	+	+	+			+	+	+	6	-614.66	27.775	0
	+	+	+				+	+	5	-613.657	28.777	0
	+	+	+				+		4	-613.074	29.36	0
	+	+		+	+	+			5	-612.054	30.38	0
	+	+		+	+	+		+	6	-611.031	31.403	0
	+	+		+	+	+	+		6	-609.826	32.608	0
	+	+	+		+				4	-609.511	32.924	0
	+	+		+	+	+	+	+	7	-608.761	33.673	0
	+	+	+		+			+	5	-608.621	33.814	0
	+	+		+	+		+	+	6	-608.01	34.425	0
	+	+		+	+		+		5	-607.839	34.595	0
	+	+		+	+			+	5	-607.574	34.86	0
	+	+		+	+				4	-605.083	37.352	0
	+	+	+					+	4	-602.883	39.551	0
	+	+	+						3	-596.308	46.126	0

Supplementary Table 3 | Forty models with the lowest AIC_c values (out of 64 models in total) explaining the ΔAUC between all pairs of the EAR_{in} and EAR_{out} curves. Each model is one line of the table. Predictors used in each model are marked by '+'. The models are ranked by their AIC_c (Akaike Information Criterion corrected for small sample sizes). The second line of the table gives standardized regression coefficients (β) for a given predictor, averaged over all of the 64 models. Green filling marks the model that is illustrated in Fig. 6b of the main text.

	Mean Moran's I of the ranges	Mean range size	Mean of sqrt(range size)/perim eter	Moran's I of richness	Richness gradient	Total richness	# of predictors	*AICc*	*ΔAICc*	*AICc* weight
Averaged standardized coefficient (β)	-0.428	-0.319	-0.011	-0.205	0.029	-0.14				
Fig. 6B	+						1	77.083	0	0.175
		+					1	78.871	1.787	0.072
		+		+			2	79.404	2.305	0.055
	+					+	2	79.561	2.479	0.051
	+		+				2	79.616	2.534	0.049
	+	+					2	79.636	2.553	0.049
	+			+			2	79.762	2.678	0.046
	+				+		2	79.79	2.708	0.045
			+				1	80.562	3.019	0.039
		+			+		2	81.124	3.478	0.031
						+	1	81.466	4.039	0.023
		+				+	2	81.47	4.381	0.02
		+	+				2	81.49	4.386	0.019
	+		+			+	3	81.767	4.406	0.019
		+		+		+	3	81.768	4.668	0.017
	+	+		+			3	81.994	4.685	0.017
		+		+	+		3	82.16	4.902	0.015
	+			+	+		3	82.251	5.054	0.014
	+			+		+	3	82.27	5.163	0.013
				+			1	82.286	5.184	0.013
	+	+	+				3	82.357	5.199	0.013
		+	+	+			3	82.443	5.275	0.013
	+	+				+	3	82.489	5.343	0.012
	+		+		+		3	82.526	5.406	0.012
	+				+	+	3	82.594	5.444	0.011
	+		+	+			3	82.607	5.511	0.011
					+		1	82.633	5.524	0.011
	+	+			+		3	82.675	5.548	0.011
			+	+			2	82.921	5.592	0.011
				+		+	2	83.121	5.833	0.009
			+			+	2	83.177	6.033	0.009
			+		+		2	83.321	6.092	0.008
		+			+	+	3	83.826	6.237	0.008
					+	+	2	84.037	6.742	0.006
		+	+		+		3	84.102	6.952	0.005
		+	+			+	3	84.449	7.017	0.005
	+		+	+		+	4	84.749	7.364	0.004
				+	+		2	84.801	7.665	0.004
	+	+		+		+	4	84.825	7.711	0.004

Supplementary Table 4 | All models explaining the ΔAUC between all pairs of the EAR and $PDXAR$ curves. Each model is one line of the table. Predictors used in each model are marked by '+'. The models are ranked by their AIC_c (Akaike Information Criterion corrected for small sample sizes). The second line of the table gives standardized regression coefficients (β) for a given predictor, averaged over all of the models. Green filling marks the model that is illustrated in Fig. 6c of the main text.

	**Inward/ Outward/ Random**	**Gamma statistic**	**Stemminess**	**Colless**	**# of predictors**	AIC_c	ΔAIC_c	AIC_c **weight**
Averaged standardized coefficient (β)	0/-1.53/-1.66	0.35	0.21	-0.027				
	+	+	+		3	145.954	0	0.731
	+	+	+	+	4	148.21	2.255	0.237
	+	+		+	3	153.373	7.419	0.018
Fig. 6c	+	+			2	153.898	7.944	0.014
	+			+	2	166.052	20.098	0
	+		+	+	3	168.004	22.5	0
	+				1	168.046	22.091	0
	+		+		2	168.64	22.686	0
		+	+		2	227.098	81.144	0
		+			1	228.599	82.645	0
		+	+	+	2	229.322	83.368	0
		+		+	1	229.75	83.796	0
					0	233.016	87.061	0
				+	1	233.388	87.434	0
			+		1	234.464	88.51	0

Supplementary Notes

Supplementary Note 1 | Scale, grain and extent of habitable area loss

For framing magnitudes of extinction with habitable area loss (using EAR) two aspects of spatial scale are important: *extent* and *grain*. The critical spatial extent for assessing extinctions is global, since a global loss is irreversible. Grain of habitable area loss can be defined as the size of the smallest contiguous blocks of habitable area that are lost; it is the smallest spatial unit of the loss which is represented by the area axis (x-axis) of EAR, and in such definition small (local) grains are again irrelevant for extinction estimates.

An alternative view is that grain is the spatial resolution at which complex shapes of immediate loss (e.g. networks of logging roads) emerge, and in such view the fine-grain configuration of loss can be critical. However, examining such configurations of loss is only possible at small extents as it requires comparably fine-grain information on species distributions.

At global extent and for the best known taxa the distributional data are only available at large grains above roughly 110×110 km (1 degree), and hence any study of empirical EARs at continental to global extents can only examine patterns of loss at grains of 1 degree and coarser.

Supplementary Note 2 | The difference between Models 3 and 4, with additional derivations, details and illustrations

Here we show, in richer detail than in the main text, that the difference between the inward and outward extinction curves produced by Models 3 and 4 is inevitable. Moreover, the two models give inverse relative positions of the inward and outward curves. Specifically, we show that Model 3 (with mid-domain effect) always gives a steeper extinction curve for the outward area loss relatively to the inward loss. In contrast, Model 4 (without mid-domain effect) always leads to a steeper or equally steep inward extinction curve, relatively to the outward curve.

Notation

In this section we use the following abbreviations and terms, which are also illustrated in Figure 2e in the main text.

R - The entire circular region that consists of the inner and outer domain.

r_r - In Model 3 this is the radius of a circular species' range that is placed entirely within the boundaries of R. In Model 4 this is the radius of a circular *potential species' range* that is placed into the region R, and possibly truncated by its boundary, so that a realized range emerges.

r_{in} - Radius of the inner circular domain.

r_{tot} - Total radius of the circular region R.

A_{in} - Area of the inner circular domain, which is $A_{in} = \pi r_{in}^2$.

A_{out} - Area of the outer circular domain (annulus), which is $A_{out} = \pi r_{tot}^2 - \pi r_{in}^2$.

P_{in} - In Model 3 this is the probability that the range with radius r_r will fall entirely within the inner circular domain. In Model 4 this is the probability that the potential range with radius r_r will produce a realized range falling entirely within the inner circular domain.

P_{out} - In Model 3 this is the probability that the range with radius r_r will fall entirely within the outer circular domain (annulus). In Model 4 this is the probability that the potential range with radius r_r will produce a realized range falling entirely within the outer circular domain (annulus).

$P_{overlap}$ - In Model 3 this is the probability that the range with radius r_r will overlap the boundary between the inner and the outer domain. In Model 4 this is the probability that the potential range with radius r_r will produce a realized range overlapping the boundary between the inner and the outer domain.

Model 3 with mid-domain effect

In this model we are randomly placing a contiguous circular range entirely within region R, so that it never overlaps its outer boundary (Supplementary Figure 4a). This model inevitably leads to what is known as the *mid-domain effect* – a higher concentration of range centroids in the centre of the region than around its boundary. Here we provide formulas for the following probabilities:

The probability P_{out} that a range with radius r_r will fall **entirely within the outer domain** is:

$$P_{out} = \begin{cases} 1, & \text{if } r_{in} = 0 \\ 0, & \text{if } r_r \geq (r_{tot} - r_{in})/2 \\ X_{out}, & \text{if } r_r < (r_{tot} - r_{in})/2 \end{cases} \tag{1}$$

where

$$X_{out} = \frac{(r_{tot} - r_r)^2 - (r_{in} + r_r)^2}{(r_{tot} - r_r)^2} \tag{2}$$

X_{out} is the ratio of the outer area A_{out} to the total area A_{tot}, both adjusted by r_r, which eliminates the possibility of the range overlapping the region R's outer boundary, and which is a sufficient condition to invoke the mid-domain effect.

The probability P_{in} that the range with radius r_r will fall **entirely within the inner domain** is:

$$P_{in} = \begin{cases} 0, & \text{if } r_r \geq r_{in} \\ X_{in}, & \text{if } r_r < r_{in} \end{cases} \tag{3}$$

where

$$X_{in} = \frac{(r_{in} - r_r)^2}{(r_{tot} - r_r)^2} \tag{4}$$

X_{in} is the ratio of the inner area to the total area, both adjusted by r_r to invoke mid-domain effect.

The probability $P_{overlap}$ that the range will **overlap the boundary between the inner and outer domain** is:

$$P_{overlap} = 1 - P_{out} - P_{in} \tag{5}$$

Model 4 ***without*** mid-domain effect

In this model the circular range (which we call *potential range*) is placed randomly into region R, it is allowed to overlap the R's outer boundary, and the part of the range that ends up outside of the boundary is eliminated (Supplementary Figure 4b). Hence, the resulting *realized range* is only the cropped part of the original range that lies inside R. The probability P_{out} that the realized range lies **entirely within the outer domain** is:

$$P_{out} = \begin{cases} 1, & \text{if } r_{in} = 0 \\ X_{out}, & \text{if } r_{in} > 0 \end{cases} \tag{6}$$

where

$$X_{out} = \frac{(r_{tot} + r_r)^2 - (r_{in} + r_r)^2}{(r_{tot} + r_r)^2} \tag{7}$$

X_{out} is the ratio of the outer area to the total area, both adjusted by r_r of the potential range to eliminate the mid-domain effect. Note that here the r_r is added, rather then subtracted from r_{tot} as in the 'mid-domain' case of Model 3. Also note that in Model 4 the centres of the potential circular ranges may lie close to, or even beyond, the boundary of R (Supplementary Figure 4b).

The probability P_{in} that the realized range lies **entirely within the inner domain** is:

$$P_{in} = \begin{cases} 1, & \text{if } r_{in} = r_{tot} \\ 0, & \text{if } r_r \geq r_{in} \\ X_{in}, & \text{if } r_r < r_{in} \end{cases} \tag{8}$$

where

$$X_{in} = \frac{(r_{in} - r_r)^2}{(r_{tot} + r_r)^2} \tag{9}$$

X_{in} is the ratio of the inner area to the total area, both adjusted by the r_r of the potential range to eliminate the mid-domain effect.

The probability $P_{overlap}$ that the range will **overlap the boundary between the inner and outer domain** is:

$$P_{overlap} = 1 - P_{out} - P_{in} \tag{10}$$

The proof: P_{in} and P_{out} as functions of range radius

In order to have the the effect of inner (A_{in}) and outer (A_{out}) areas fully under control, and hence to focus fully on the difference between the inward and outward direction of destruction, we set $A_{in} = A_{out}$. Under this condition it follows that $r_{in} = r_{tot}/\sqrt{2}$, which allows us to calculate P_{in}, P_{out} (equations 1, 3, 6, 8) and $P_{overlap}$ for any r_r.

For the total number of S_{tot} species that live in the region R, and are indexed by i, it follows that $E_{in} = \sum_{i=1}^{S_{tot}} P_{in_i}$ and $E_{out} = \sum_{i=1}^{S_{tot}} P_{out_i}$. In other words, to get the mean number of species E_{in} that go extinct with the loss of A_{in} we need to sum up the probabilities P_{in_i} for all species that live in the region R. Thus:

- For **Model 3 it always holds that $P_{in} \geq P_{out}$, from which follows that $E_{in} \geq E_{out}$**. This holds for any range size and for any imaginable frequency distribution of range sizes, as the curves in Figure 2f involve all possible range sizes (represented by r_r).
- **Model 4** is somewhat trickier since the potential circular range can be truncated by R's boundary, and hence the r_r of the potential range is not directly proportional to the area of the realized range inside R (Supplementary Figure 4b). Figure 2g shows that, for potential ranges with $0 < r_r \leq r_{tot}$, $P_{in} < P_{out}$ and hence $E_{in} < E_{out}$. Supplementary Figure 6 extends this reasoning for potential ranges with $r_r > r_{tot}$, showing that $E_{in} < E_{out}$ holds for potential ranges with r_r roughly up to 100× the size of r_{tot}; above that threshold we see that $E_{in} \approx E_{out}$ (Supplementary Figure 6). This means that **any imaginable frequency distribution of potential range sizes in which at least one potential range satisfies $0 < r_r < r_{tot} \times 100$ will lead to $E_{in} < E_{out}$, and $E_{in} \approx E_{out}$ otherwise**[1].

Probability that the range is lost as a function of lost area

To further illustrate the discrepancies between Models 3 and 4 we can use equations 1, 3, 6 and 8 to plot the probability that a randomly placed range will be lost (P_{in} or P_{out}) as a function of lost area (A_{in} or A_{out}) – this contrasts with the previous section where $A_{in} = A_{out}$. We also set $A_{tot} = 1$, leading to $r_{tot} = \sqrt{1/\pi}$.

- In case of the **outward area loss** we eliminate the inner circular domain and calculate the probability that the species range occurs entirely within the inner domain; hence, for a given range perimeter r_r, we plot P_{in} (from equations 3 or 8) against A_{in}.
- In case of the **inward area loss** we eliminate the outer domain (annulus) and calculate the probability that the species range will occur entirely within the outer domain; for a given range perimeter r_r we plot P_{out} (from equations 1 or 6) against A_{out}.

We have chosen to plot the curves for r_r of 0.01, 0.1 and 0.5 (Supplementary Figure 7), and in all three cases the results are consistent with the simulations (Fig. 2c-d): In Model 3 the outward area loss leads to higher probability that a species is lost, while the opposite holds for Model 4.

[1]Now let us stay in the extremely simplified scenario of Model 4, but let us set the sizes of the circles in line with the dimensions of the real World. The largest imaginable range size of a terrestrial species is the area of all landmass, which is 148, 939, 063.133 km^2. The area of our rectangular regions is 484, 0000 km^2 (Supplementary Figure 1). This gives the maximum possible ratio r_r/r_{tot} to be 5.69; which very clearly gives $P_{in} < P_{out}$ according to Supplementary Figure 6.

Supplementary Methods

Functional traits used to calculate functional diversity

The trait categories were composed of single (body size, activity time) or multiple variables (diet, foraging niche).

Diet was characterized as proportional use of each of seven dietary categories for mammals (seeds, fleshy fruits, nectar and pollen, other plant material, invertebrates, fish, vertebrates) and eight dietary categories for birds (seeds, fleshy fruits, nectar and pollen, other plant material, invertebrates, fish, carrion, other vertebrates).

We used **activity time** as an ordinal variable with five categories: (1) nocturnal, (2) nocturnal and crepuscular, (3) crepuscular or cathemeral, (4) diurnal and crepuscular, (5) diurnal.

To represent variability in **daytime activity patterns** we matched bird foraging height data to that of mammals in the form of four ordinal categories: (1) ground level, (2) scansorial or low vegetation or understory, (3) fully arboreal or canopy, (4) aerial.

Finally, we classified the species by the **environment in which they forage** to: (1) aquatic, (2) semi-aquatic, (3) terrestrial or non-aquatic.

For additional details see Supplementary References 1 and 2.

Details of the calculation of the regional predictors

- **Gamma statistic** γ. We used function `gamStat` in R package `laser` to calculate the γ.
- **Colless index**. We calculated the index by using the `colless` function in R package `apTreeshape`.
- **Mean Moran's I of the ranges**. We measured the global Moran's I [3] using function Moran in R package `raster` (using the default 3×3 'King-style' neighborhood). We note that this measure will always be inevitably correlated with average range size (Supplementary Table 1), and hence a region with high mean Moran's I of the ranges will also have large mean range area.
- **Richness gradient**. We created map of species richness for each taxon in each region. We then took geographic coordinates (latitude and longitude) of each grid cell and calculated an ordinary least squares regression (function `lm` in R) of the richness at the cells against latitude and longitude of the cells, and their interaction. In the R-style formula specification the model would be written as: `lm(richness ∼ latitude + longitude + latitude:longitude)`. We then calculated R^2 of this regression, which is our index of richness gradient.
- **Moran's I of richness**. We measured the global 1st distance class autocorrelation of cell-specific values of species richness by the global Moran's I (using the 3x3 'King-style' neighbourhood). We used the function `Moran` in R package `raster` for that.

Pearson correlations among the variables are given in Supplementary Table 1.

Details of the statistical models explaining AUC and ΔAUC

We used ordinary least squares regression (OLS; Normal error distribution) implemented in function `lm()` in R. We used R functions `dredge` and `model.avg` (package `MuMIn`) to perform the multiple model comparison and averaging.

Note: Here we mean the statistical models fitted to the empirical data and *not* the four theoretical models of range placement.

Supplementary References

1. Wilman, H. J. *et al.* EltonTraits 1.0: Species-level foraging attributes of the world's birds and mammals. *Ecology*, **95**, 2027 (2014).

2. Belmaker, J. & Jetz, W. Cross-scale variation in species richness–environment associations. *Glob. Ecol. Biogeogr.*, **20**, 464–474 (2011).

3. Legendre, P. & Legendre, L. *Numerical Ecology*, Elsevier (2012).
